# Diverged Preferences towards Sustainable Development Goals? A Comparison between Academia and the Communication Industry

**DOI:** 10.3390/ijerph16224577

**Published:** 2019-11-19

**Authors:** Shin-Cheng Yeh, Haw-Jeng Chiou, Ai-Wei Wu, Ho-Ching Lee, Homer C. Wu

**Affiliations:** 1 Graduate Institute of Environmental Education, National Taiwan Normal University, Taipei 11677, Taiwan; awu@ntnu.edu.tw; 2 Department of Business Administration, National Taiwan Normal University, Taipei 10645, Taiwan; hawjeng@ntnu.edu.tw; 3 Graduate Institute of Construction Engineering and Management, National Central University, Taoyuan 32001, Taiwan; hoching@ncu.edu.tw; 4 Graduate Program of Sustainable Tourism & Recreation Management, National Taichung University of Education, Taichung 40359, Taiwan; hcwu@mail.ntcu.edu.tw

**Keywords:** sustainable development goals (SDGs), health and well-being, inequality, academic publications, the media industry

## Abstract

To understand how the sustainable development goals (SDGs) are involved and cited in different fields, the current study aimed to explore the key SDGs and SDG-pairs from the viewpoints of academia and the media. The academic publications with SDG(s)-related keywords in the Scopus database and the entry videos of the “2018 SDG Lions” were collected and analyzed through content and network analysis. It was found that SDG 3 and SDG 10 shared the highest preferences in both industries, but apparent gaps happened to SDG 5. The tied frequencies of the possible SDG pairs were also examined, and SDG 3-10 was identified taking the lead in both industries. Network analysis using degree centrality as the vital parameter demonstrated that SDG 8 and SDG 5 has strong connections with several SDGs for the academia and the media, respectively. The SDG-2-6-7 combination or “water-energy-food” nexus was also found the most frequent combination of three SDGs in the academia. Overall, SDG 3 can be treated as a unifying theme when seeking to acquire evidence-based knowledge for integrated implementation of the SDGs. Important implications for policy-making of the SDGs were also discussed.

## 1. Introduction

There is a long historical tradition for the United Nations (UN) to advocate global partnerships for achieving the sustainability of people and the planet. Three essential missions of the UN systems sustaining global actions for sustainable development include agenda-setting, consciousness-raising, and strategy-deciding since the Earth Summit in 1992 [1,2]. In 2015, the UN adopted 2030 agenda of the Sustainable Development Goals (SDGs) including 17 goals with 169 targets in the second level and claimed the balanced importance among triple bottom lines (TBL), i.e., economic development, social inclusion, and environmental protection, for all countries from 2015 to 2030 [3]. Thus, it is useful to gain evidence-based knowledge among the global communities when intending to implement the SDGs in an integrated way [4].

Different analytic methods towards different SDG-relevant data were applied to clarify the relationships between and among SDGs [1,5]. In a UN Department of Economic & Social Affairs (DESA) Working Paper, Le Blanc envisioned SDGs as a network of targets and thus employed network analysis to find out their interrelationships. Secure connections were identified between some SDGs, like SDG 4 and 5, SDG 1 and 10, and SDG 10 and 16. Besides, he found SDG 12 is the goal with most linkages to other SDGs [1]. Pradhan et al. (2017) used the official data of the SDG indicators of UN Statistics Division 2016 and did Spearman’s rank correlation to examine the significant characteristics of the relationship between two sustainable development goals or two targets under the same goal. The top two synergy interlinkages of “SDG 3–SDG 6” and “SDG 3–SDG 5” on a global scale were found [5]. Nilsson et al. argued that conceptually, once the features of essential interactions among the SDGs were identified, they would be employed by the policymakers for planning reasonable priorities of SDGs implementations and for avoiding negative trade-offs and creating positive synergies in terms of evidence-based knowledge [6,7,8].

Apart from the discussion of methodology, stakeholders’ interests also potentially influence the integrated implementation of SDGs. The academic community has actively explored critical SDG linkages. Some papers have focused on understanding the associations between one core SDG and other SDGs [9,10,11,12,13] while others have explored meaningful nexus among SDGs [14,15,16], or even elaborated the interlinkages among all goals or targets as mentioned above [1,5,17]. Regarding the SDG 14 (life below water) as a core concern, Singh et al. explored the social-ecological context, the compatibility feature and the prerequisite of interactions. They found that establishing marine protected areas (SDG 14.5) could negatively influence improving rights and access to resources (SDG 1.4) if areas are established and enforced without proper consultation with local stakeholders [11]. Researches exploring the multiple interactions of Water-Energy-Food (WEF) nexus, namely the interconnections of SDG6-SDG7-SDG2, are burgeoning [18,19]. For example, in 2015, both the National Natural Science Foundation of China (NSFC) and US National Science Foundation (NSF) either prioritized WEF nexus research area or initiated WEF nexus program [19]. The meaning of SDG6-SDG7-SDG2 nexus discloses the importance of the security of water-energy-food with the thriving population and consumption levels. However, focused domain differences exist. Endo et al. reviewed the current state of research on the water-energy-food nexus and found the most significant number of publications were associated with the linkage of water-energy (32%) [20]. Regarding influential international organizations, the World Economic Forum framed ‘water’ as the more influential domain than the others [21]; the Food and Agriculture Organization valued food security and sustainable agriculture beyond the other two [22]. Overall, Leal Filho et al. pinpointed the UN SDGs might motivate sustainable development (SD) researchers in interdisciplinary areas to generate integrated knowledge and in turn benefit policy-making [23] although currently there were gaps in the judgement of synergy or trade-off relationships between targets [24]. 

To mainstream the 2030 agenda worldwide, the establishment of global awareness was the first step [25]. All governments were called by the UN to work on this but also were business/industry communities involved. Several months after the launch of the SDG in late 2015, the Secretary-General of the UN together with the CEOs of the six most prominent companies in the marketing and communication industry made a firm promise to support the SDGs at the 2016 Cannes Lions Festival of Creativity. The initiative was called the “Common Ground” as the whole industry was asked to commit themselves and act [26]. Besides, to create institutional incentives and to reward real impact in SDG-related actions, the awards of the sustainable development goals of Cannes Lions (so-called SDG Lions) were formed and announced in UN General Assembly in 2018, as a follow-up of the events in 2016 Cannes Lions Festival of Creativity. The global communicative talents were invited to make the world better by connecting their social innovation ideas to the SDGs. With the support of the leading marketing and communication industries and participation of private and public sectors around the world, there were 687 entries for this event. Among them, 20 works got the SDG Lion prizes. The first prize of this historical event granted the work “Palau Pledge,” describing Palau’s national action in revising tourism rules for environmental conservation. The real impacts of “Palau Pledge” were not only the transformation of tourism experience and immigration processes but global awareness in intergenerational justice in terms of environmental quality and biodiversity. As the concluding remarks of that video presented: “A mother hopes her children can see the beautiful place as her see today.” The SDG Lions was a meaningful platform for communicating and promoting the concepts and practices of sustainable development. As it has been discussed extensively that the academic research cannot quickly impact the general public and associated policies, the design of the SDG Lions showed both rational reward and sentimental justice discourse representing the offer of incentive structure in conservation [27]. Besides, to engage the non-scientists with unfamiliar and elaborate information, ICT-based visualization can be of help [28,29]. The participants of the SDG Lions were encouraged to apply all kinds of communication and marketing strategies and applicable technology like animated figure and high-impact statistics in their videos. Notation of sustainable development and linkages of potential practices and the SDGs were integrated into the media representations with arranged information and affection inducing design made the SDG Lions an alternative way of communication when compared to the academia. 

There seems to be a lack of research to explicitly compare the focused SDGs between the academia and the media (communication industry) although both are the main strategies in disseminating messages. For instance, Edwards et al. represented the top information-providing strategies of health knowledge translation should include both journal publications (in second place) and media campaigns (in fourth place) [30]. For some knowledge and resource-intensive topics, the distribution of various communication or marketing schemes may be more skewed. Endo et al. studied identified stakeholders in the NEXUS Resource Platform and 37 projects and found over 50% of the stakeholder organizations belonged to the research field but less than 1.5% to the media [20]. The current study tried to supplement and analyze that phenomenon of significant but absent communication industries towards the understanding and spread of SDG-related concepts. Besides, to know the issues the communication industries concern about and to clarify the differences and similarities of emphasized SDGs between the academia and the media industry would be vital for the integrated implementation of the 2030 agenda. Furthermore, there was a lack of research in exploring the interactions of 17 SDGs of global academic publications via the quantitative methodology employing content and network analysis techniques. Take the study of Körfgen et al. as an example: projects and publications represented by universities were analyzed only in Austria [31]. Besides, Salvia et al. surveyed the SDGs receiving more attention among academic staff globally [32], in which snowball sampling might oversample a particular network of academic peers [33]. Based on the curiosity towards the share of each SDG’s voice in professional publications and the entries of SDG Lions, the current study intended to clarify the trends of the academia and the media with SDG-related topics and the interactions of SDG pairs via content and network analysis. 

Academic experts and the mass media are two key groups of stakeholders when implementing the SDGs. Specifically, SDG-related policies would be proposed by academic experts based on the results of academic researches. However, wide-spread recognition and effective communication of these policies need the support of the media. Thus, it is crucial to elucidate the similarity and difference of opinions toward the SDGs between those two industries. Based on the data from academic references in Scopus and the entry and award lists of 2018 SDG Lions, the current study sought to understand the focused SDGs in academic and communication industries and further explore the relationships of the common SDG and other SDGs between two industries for further examination of integrated SDG-related actions. 

## 2. Materials and Methods 

The present study aimed to explore the similarities and differences of emphasized SDGs between the academia and media for the sake of understanding the viewpoints of SDG-related stakeholders and the potential resulting coordination and integration. The power of academic experts and mass media on policymaking has long been recognized. Lindblom thought policy-making is the consequence of processes in the participation of various specialized elites [34] and McCombs and Shaw claimed mass communication could influence people’s opinion on agenda development [35]. The SDG-related academic publications from 2015 to 2018 in the Scopus database were selected to represent the sources in academia. On the other hand, the entries and award lists of the SDG-promoting videos of the 2018 Lions in “THE WORK” website were used for presenting the focuses of the media. Content analysis and network analysis was used to find out the influential SDGs and SDG combinations. Therefore, the current study would analyze the features of the academic publications for each SDG annually and totally from 2015 to 2018, as well as the preference of communication communities in 2018 SDG Lions for the first step for comparing the influential SDGs between the two industries. Furthermore, the strength of the SDG pairs with the most influential SDG as the core was also analyzed. From a holistic perspective, the degree centrality for network analysis was calculated in this study to know the power of each SDG in the 17-goal network. Finally, the multiple connections of SDGs in the academic publications were also checked.

**Figure 1:**
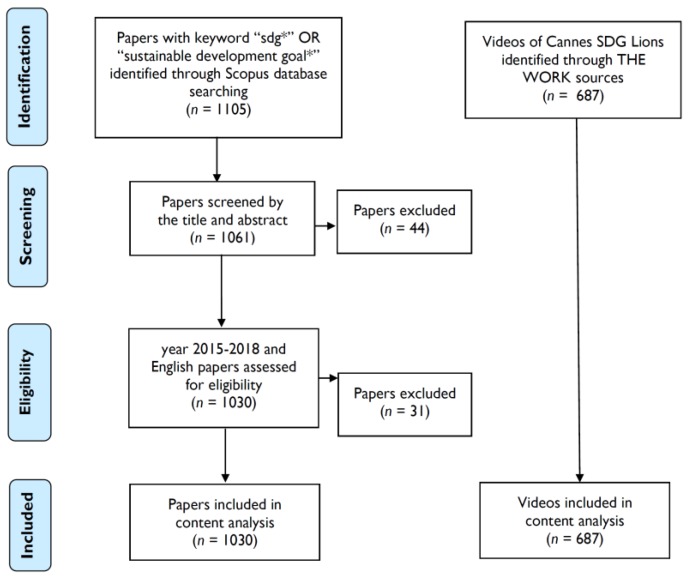
The flow diagram of the present systematic review study.

The content analysis could function as clarifying the context of text corpus quantitatively. As the UN has already well defined the 17 SDGs in official documents, these are suitable as the coding frame for academic publications because the problem of researchers’ prejudices can be avoided. Besides, based on the coding frame of those 17 SDGs, originators of videos in 2018 SDG Lions directly attributed SDG(s) to each entry. Thus, it is not necessary to address the procedure of inter-rater reliability in the SDG(s) category. Several steps for content analysis were suggested by Bauer, including the selection of particular texts, sampling, coding frame construction, defining the coding rules, reliability test, process reliability establishment, data file setting, and statistical analysis [36]. For this study, academic publications were selected in terms of the keywords of “sustainable development goal* or sdg*” in the Scopus database first. These were made use of as the source of the SDG-related references in some studies such as McCollum et al. and Albrecht et al. [10,18]. There were 1105 peer-reviewed publications included at the initial stage. Next, to explicitly define the coding rules for each frame, the current study referred to the supplement in the research of McCollum et al. as the foundation of coding rules [10]. After browsing the keywords offered in the Scopus database, those rules were iteratively expanded and revised into coding and saturation status. The condition of the same rule in different SDGs was avoided. For example, although SDG 5 and SDG 10 both involve the issues of inequality, only gender-related terms plus equality can be attributed to SDG 5. Finally, 1030 publications in English were sieved out from 1,105 papers because papers with “sdg” as a keyword but irrelevant to sustainable development goals were removed at this stage (see Figure 1).

At the coder training section, all the coding frames and rules were provided (see as Table 1), and ambiguous examples were answered. Seventeen SDGs (coding frames) were independently coded for each paper as 0 or 1. Each coder was given coding instructions, the overlapping references, and coding spreadsheets in separate electronic files. For the whole process, moderate inter-rater reliability (Cohen’s kappa coefficient =0.46 in SPSS 23 (IBM, Armonk, NY, USA)) was obtained [37]. Bauer thought there are several research designs of content analysis and the current study applied both longitudinal and cross-sectional analysis [36]. The methodology mentioned above can be used to support both analyses, especially the changing SDG focuses on academic papers from 2015 to 2018.

In addition to the relevance frequencies of the respective SDGs, the tied frequencies between a pair of two SDGs were also of significant concern. In network analysis, the symmetric one-mode matrix of a total number of 136 (17*16/2) SDG pairs can offer a holistic view among them [38].

Frequencies of the co-occurred two SDGs were calculated through EXCEL (Microsoft Office 2016, Redmond, WA, USA) and then graphed using the network analysis software UCINET 6.0. UCINET (Analytic Technologies, Harvard, MA, USA), representing the University of California at Irvine NETwork, introduced by Freeman and maintained by Borgatti and Everett. The network analysis technique used here is often applied for the studies of sociology, management, politics, and other fields [38]. The process of the study included coding frame and rules, data collection, data coding and reliability, and statistical analysis.

## 3. Results and Discussion

### 3.1. Annual and Total Features of Academic Publications for Each SDG

**Figure 2:**
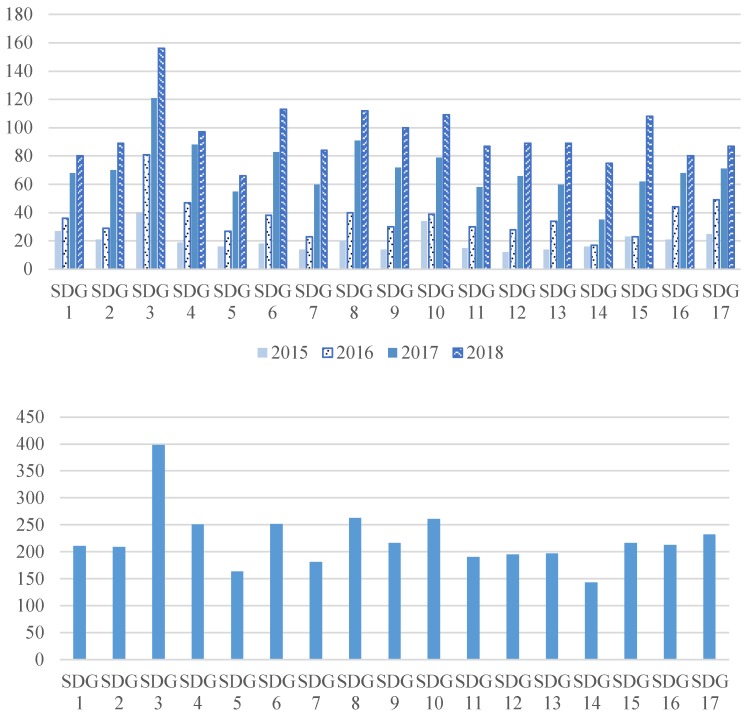
The number of academic references for each SDG in each year (upper) and the sum from 2015 to 2018 (lower).

To understand the most influential SDGs in academic communities at different time intervals, we applied content analysis for gaining the frequency of each SDG mentioned in academic publications. Relevant information was shown in Figure 2. Apparently, the amount of SDG-related academic papers have been continuously increasing at an individual SDG level. The most common SDG-related topics fell in SDG 3 (health and well-being), SDG 8 (economy and work), SDG 10 (reduce inequality), SDG 6 (water and sanitation), and SDG 4 (quality education) for the sum in the four years.

Notably, the top status of SDG 3 was reaffirmed at all time intervals. On the other side, the least connected SDGs were SDG14 (life below water) and SDG5 (gender equality) for academia. Also, SDG 12 at the least rank in 2015 became rank 8th in 2018. Finally, the amount of SDG 3, SDG 6, and SDG 8 in 2018 have a considerable increase compared to those in 2015.

According to the definition of health from the World Health Organization (WHO), health is “a state of complete physical, mental and social well-being and not merely the absence of disease or infirmity.” [39] This means that health involves the satisfaction of various human basic needs. Maslow proposed a theory regarding human motivation and thought the hierarchy of basic human needs are from physiological and safety needs to love, and esteem needs even the need for self-actualization [40], which seem to respond to the definition of health made by WHO. Thus, seeking to and maintaining health even avoiding unhealthiness might be a human basic needs and policy advocacy with public health implication was readily accepted by the public and further mobilized the public to support that policy [41,42]. After Rio+20, the voice of “the right to health” were amplified by health professionals Hill and others via publications at the moment of agenda-shifting [43,44,45]. Additionally, the promotion of influential groups like the World Health Organization (WHO) also could be the main factor of plenty of the SDG-related researches involving SDG 3 [46,47]. SDG 3 represented diverged meaning between the experts in the research of Salvia et al. (2019) and the intended discourses of experts in SDG-related, especially health and welfare, research works [32]. It was also found in recent studies that SDG3 was among the SDGs having most synergies with others [9] or having synergies in most countries [5,48].

### 3.2. The Preference of Communication Communities in 2018 SDG Lions

**Figure 3:**
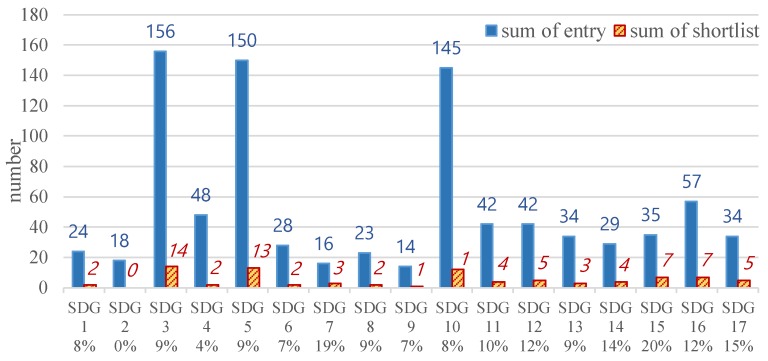
The proportion of each SDG’s entries (blue bars and numbers) to shortlists (orange line and numbers) in 2018 SDG Lions.

The UN recognized the importance of spreading SDG toward the global public at the initial stage, as mentioned in the preceding sections. The SDG Lions has the symbolic meaning in the media communities worldwide. The current study explored the features of each of the 687 SDG entries in 2018, the first SDG prize in Cannes Lions Festival. It tried to discover the most common SDGs emphasized by creativity communication industry. According to the results shown in Figure 3, SDG 3 (health and well-being) and SDG 10 (equality), just like the academia, shared the highest preference in media communities, whereas SDG 5 (gender equality) was only promoted in 2018 SDG Lions. It is not difficult to understand this as gender equality issues have been widely discussed and represented in the media. It can be observed through Figure 3 that the proportion of entries (reflecting the preference of originators) to awards (reflecting the preference of judges) seem to be consistent. However, compared with the percentages (8 or 9%) of entries to awards in top three referred SDGs, some SDGs won additional supports by the judges of 2018 SDG Lions, such as SDG 15 (life on land) and SDG 7 (clean energy). On the other side, SDG 2 (hunger and nutrition) did not enter the shortlist of 2018 SDG Lions even though the number of entries in SDG 2 was not the lowest. There might be some preference for the judges of 2018 SDG Lions towards some specified SDGs.

In the five prize winners related to SDG 5 (gender equality), four videos voiced explicitly for women and one for the gender minority. In the pro-women videos, half viewed women as victims under the threat of child marriage and domestic violence while the other half recognized women’s right and capacity in stewardship and the political arena. According to the developing history of feminism, the award-winning videos represent the characteristics of both the second- and third-wave feminism. The images of help-needed and strong women were categorized into the second and the third wave, respectively [49]. 

UN Women Goodwill Ambassador Emma Watson, a young British actress famous for the role of a know-it-all girl in the movie “Harry Potter”, claimed that ‘No country in the world can yet say they have achieved gender equality’ and called for men to promote gender equality at United Nations Headquarters [50]. In 2015, many famous women in the global entertainment industry altogether voiced for gender equality. Take the senior movie star Meryl Streep for example. She has urged the US Congress to back the equal rights amendment on the purpose of guaranteeing a balance of power for women under the law [51]. Across the Atlantic Ocean, a political party—Women’s Equality—formed in the UK in 2015 produced an eminent video “I’m out of office for equal pay” which was not only a material for women movement in earning equal pay but an award-winning video in 2018 SDG Lions. Thus, media marketing movement and the campaign could be a trend for equal rights movement in the digital age [52,53].

### 3.3. The Comparison of Influential SDGs between Academia and Communication Industries in 2018

**Figure 4:**
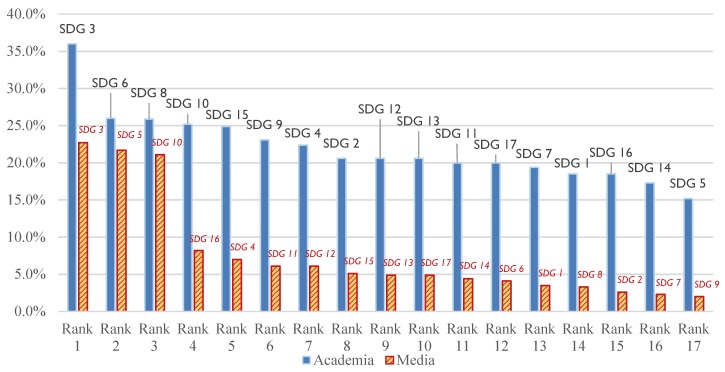
The comparison of academic (left bar) with communication (right bar) industries about the relevance percentage of the top 10 SDGs in 2018.

In 2018, 433 academic publications and 687 videos of SDG Lions were collected and analyzed. For comparison purposes, the relevance percentage of each of the SDGs, i.e., the number of related papers/videos divided by the total number of paper/videos, were calculated. Six out of the ten top ten SDGs were the same for the academia and communication industry, which means sixty per cent of consistency for those two kinds of works (please see Figure 4). Coincidentally, SDG 3 (health and well-being) was the champions in both industries. Similar to the discussion in the preceding, health and well-being representing the basic needs of people could be one of the reasons. For academia, research works were demanding for social, practical, and commercial reasons. On the other hand, health and well-being are among the vital social issues suitable to be represented as stories in the media. Besides, there were consensuses for the category of certain SDGs according to several pieces of research [54,55,56,57]. For example, unlike SDG 4, SDG 5 and SDG 16 falling into the social dimension, SDG 8 and SDG 9 expressed themselves in the economic category. In the present study, compared with economy-prone SDG 2 (hungry and nutrition), SDG 6 (water and sanitation), SDG 8 (economy and work) and SDG 9 (industry, innovation, and infrastructure) in the academia, society-prone SDG 5 (gender equality), SDG 11 (sustainable city), SDG 16 (peace and just institution), SDG 17 (global partnership) for communication community were in the top 10 frequency ranks of SDG-related works. Notably, the relevance percentage of SDG 5 in 2018 SDG Lion was much higher than that in academia. (please also see Figure 2 and Figure 4). 

Furthermore, the relevance percentages of SDG 9 (infrastructure and research) and SDG 8 (economy and work) in the academia were about ten-time of those in the media, showing a differentiated preference on these relatively “hard” topics. Academic publications are mostly produced by scholars in higher education. Although higher education was expected to cultivate citizens with global views and wisdom, it is difficult to deny that one of the purposes of pursuing higher education would be the quality workforce for good jobs. That is especially true at the age of knowledge/data/information-based economy. For example, Ford thought that one of the primary functions of higher education in American serves the national economy [58]. University students in the UK thought their higher education degree could not guarantee adequate income as the labour markets were becoming more and more competitive. However, they still agreed that higher education offered them opportunities to add value to themselves [59]. Thus, it would be reasonable to find many SD-related academic papers having connections to “decent job and economic growth” as defined by SDG 8. To sum up, even though there were some diverged perspectives, the leading status of SDG 3 (health and well-being) has achieved a consensus in both industries.

### 3.4. The Frequency Analysis of Tied Pairs in the 17-goal Network

**Figure 5:**
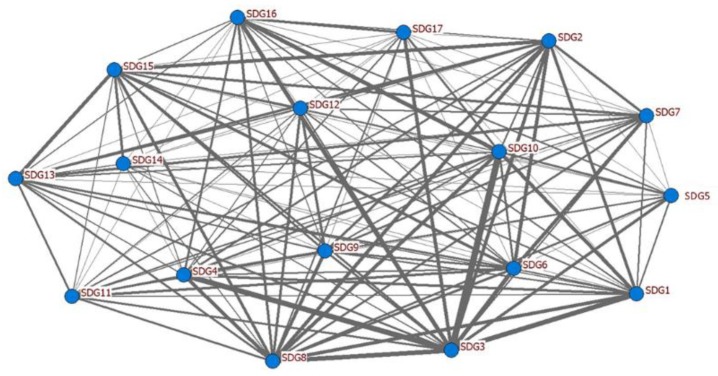
The elaborate network graph of 17 SDGs (generated using UCINET).

In addition to the relevance frequencies of the respective SDGs, the tied frequencies between a pair of two SDGs were also of significant concern. The tied frequencies of the academic publications and informative videos of SDG pairs and the total numbers of frequency for all SDGs were demonstrated in Table 2. It can be observed that SDG 3 and SDG 10 had leading frequencies among the 17 SDGs. The frequencies of the SDG pairs in academia were between 81 and 160; whereas those in the media were between 0 and 39. The overwhelming interconnections between SDG 3 and SDG 10 were reflected by the highest frequency of 160 among all possible pairs in the academia. For the media, the highest frequency between two SDGs was 39, corresponding to the pair SDG 5 and SDG 10. On the other hand, some SDGs were not involved in academic publications frequently. For example, the SDGs pair 14-17 and 5-14 had the lowest tied frequencies, which indicated research papers with the topics related to life below water (SDG 14) together with global partnership (SDG 17) or gender equality (SDG 5) being relatively fewer. In general, health and welfare issues (SDG 3) had high involvements with other SDGs no matter in the academia or media industries. These numbers can be demonstrated as a network graph created by UCINET, shown as Figure 5, in which all nodes (SDGs) were connected by linkages with different thicknesses, indicating the tied frequencies between two SDGs. According to Le Blanc (2015), seventeen SDGs should be a theoretically interconnected network [1]. In network analysis studies, the concept of “centrality” and related measurement techniques are popular. Several types of centrality measure defined by Freeman (1979) have been the widely used terminologies in the field of network analysis [60], including degree centrality, betweenness centrality, and closeness centrality [61]. In this study, as the network was relatively simple, containing 17 SDGs as the nodes connected, degree centrality was chosen as the critical measure for representing the relative degrees of connections of the nodes in the network, as explained in some pieces of literature related to graph theory and social networks [62,63]. In general, for a network with n nodes, the degree centrality in this study can be defined and formulated as follows:

(1) $$DC_{i}=\frac{\bar{C_{i}}}{\underset{\forall i\;and\;j}{max}X_{ij}}$$

where *DC_i_* is the degree centrality of the node *i*;

*X_ij_* is the number of linkages, between node *i* and node *j*:

*i*, *j* = 1 to *n*, *i* ≠ *j*; (2)

and $\bar{C_{i}}$ is the average number of linkages for all possible *n* − 1 pairs for node *i*, thus:

(3) $$\bar{C_{i}}=\frac{\sum_{j=1}^{n}X_{ij}}{n-1}$$

In this study, the “degree centrality” of each SDG in the network means the ratio of average tied frequencies incident upon it (the numerator in Equation (2)) to the most considerable tied frequency among all possible SDG pairs in the whole matrix (the denominator in Equation (2)). A higher degree centrality means the SDG had more connections/linkages with other SDGs. The top ten SDGs were shown in Table 3. Because network analysis often is applied for social science, it helps to clarify the power structure of actors. Here, each SDG as a conceptual actor is manipulated by the academic or communicative community. Thus, SDG 3 and SDG 10 were the most important goals within the network and possessed the support of both the academia and the media industries.

**Figure 6:**
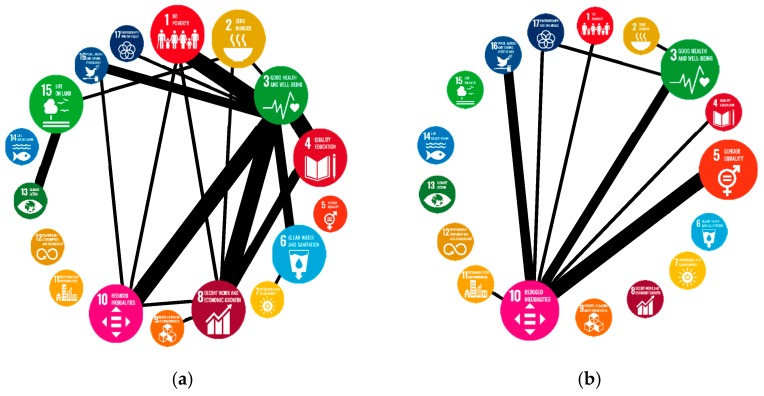
The network graph is showing SDGs and connections with higher tied frequencies of the academic publications (**a**) and media videos (**b**) of SDG-related topics from 2015 to 2018, bigger icons and thicker linkages indicating higher total frequencies of SDG and higher tied frequencies between two SDGs, respectively.

To present the essential nodes and linkages more explicitly, we redrew the network graph as Figure 6, in which several SDGs with higher total frequencies were shown as bigger icons and only those linkages with tied frequencies bigger than 125 for academia and 5 for media were shown. Thicker lines indicated higher tied frequencies. General speaking, SDG 3 and SDG 10 had powerful connections with other SDGs in both industries. Besides, SDG 8 and SDG 5 had relatively strong connections with several SDGs for academia and media, respectively.

### 3.5. The Correlation Strength of Each of the SDG 3 Pairs

**Figure 7:**
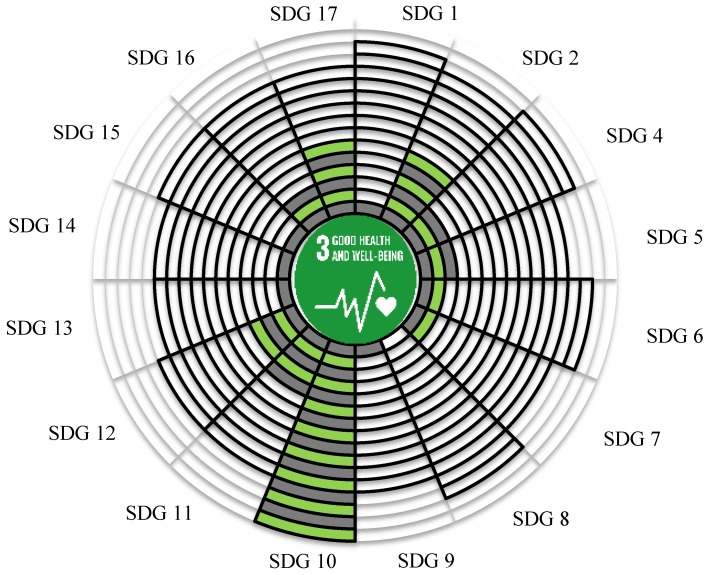
The comparison of academic (black frame) with communication (filled colored grids) industries on the tied frequency of SDG 3 and other SDGs in 2018.

SDG 3 (health and well-being) seems to be the most promoted SDG after the adaptation of the 2030 agenda no matter in the academia or the media in terms of the analysis conducted in the present study. The tied frequencies of the SDG pairs were the focuses of this study. For demonstrating the whole picture of the information for a specific SDG, a graph capable of showing the relative scales of the parameters was developed. Figure 7 was thus the graph with SDG 3 as the core theme. The proportional tied frequencies of each SDG 3 related pairs for the academia and the media were shown in different scales in the 16-grid concentric circles. The black frames and the green filled colored grids were used to indicate the tied frequencies of the SDG pairs in the academia and the media, respectively. The adjusted mean, corresponding to the range between 0 and 16, was 12.7 grids for the academia and 3.4 grids for the media.

According to Figure 7, there is a common ground in the issues of health and well-being (SDG 3) and inequality (SDG 10) from the two industries’ viewpoint. Conversely, the vast difference between the two industries happened in the frequency of SDG 3–8 linkage because it was ranked top 2 in academia but the least rank in media. Specifically, the combination of “health and well-being” and “economic growth” was never shown in a single video for the SDG Lions, but 59 academic papers related to both issues were found. Similarly, the linkages of SDG 3 and SDG 1 were also acknowledged in academia but not in the media. Because SDG 8 (economic growth) and SDG 1 (poverty reduction) both implicate finance, it seems that “health and well-being” often links to money issues in terms of the academic viewpoints. Moreover, proportional tied frequencies were clustered and left-skewed for academia but were scattered and mostly low in the media, as shown in Figure 7. Nevertheless, the issues of “health and well-being” and “equality” were well agreed with the two communities.

The World Health Organization (WHO), as the UN specialized agency for health, listed health-related SDGs and only SDG 9, SDG 14, and SDG 15 were excluded [64]. In this study, the accumulated amount of papers connecting SDG 3 and SDG 10 could be reasonable because of the endeavours from many scholars. Hosseinpoor et al., serving for WHO, indicated health inequities are “avoidable differences in health” owing to discrimination or unreachable resources. They thought health equity is a priority among the health-related topics in 2030 agenda [46]. In addition to the power from influential WHO, health experts like Hill et al. amplified voices in advocating the importance of health equity by intensively publishing papers at the year 2014 of the paradigm-shifting moment [43,44,45]. To allocate resources within the disadvantaged populations of unmet health need, no matter the universal health coverage (UHC) at a national level [45] or the health inequality monitoring systems (SDG17.18) in WHO [46] both could inform equity-oriented health strategies for high-level political decisions.

To implement integrated actions, Nunes et al. illustrated the synergy relationships between SDG 3 and other SDGs by sectors. They thought, by the cooperation of social and economic sectors, reducing inequality can result in the reduction of morbidity and mortality [65]. Besides, Nilsson et al. exemplified how the critical factors of geographical context, time horizon and governance influence the interactions among SDGs [8]. Reproductive health (SDG 3) could be promoted by SDG 5 (Gender Equality) when there are reputable institutions (governance), especially in the places of low women’s status (geographical context). The effects of different time scales also needed to be taken into consideration in associated institutions. Thus, for the relationship of reproductive health and gender equality, possible institutions in the short term could be free and quality childcare service and planned birth; in the medium- to long-term could be the supply of friendly working environment and the promotion of women’s continuing education. They also thought inappropriate governance could even counteract positive interactions among the SDGs [8]. Therefore, “health and well-being” (SDG 3) could be a unifying theme [65] when seeking to acquire evidence-based knowledge in the integration of the SDGs [66].

### 3.6. The Multiple Connections of SDGs in Academic Publications

As the 17 SDGs were interconnected in multiple ways as examined by many studies, there could be more than two SDGs related to an academic paper or a video. We set our focus on those papers with three SDGs linked for understanding the trend of integrated research and implementations. Thus, those papers associated with more than six SDGs were treated as review papers and screened out. As a total of 319 papers were identified and analyzed for finding out possible combinations of three SDGs. Table 4 listed the top combinations of three SDGs co-linked to a paper, with total frequencies more than 10. The top five included SDG 2-6-7 (food-water-energy), SDG 3-10-16 (health-inequality-justice), SDG 1-3-10 (poverty-health-inequality), SDG 3-4-10 (health-education-inequality), and SDG 1-3-4 (poverty-health-education). SDG 3 was included eight times in the top 10 list and four times in the top 5 list, demonstrating its key importance again in linking other SDGs. The high inclusion frequencies of SDG 3 and SDG 10 in Table 4 was consistent with the results for the single SDG and SDG pairs.

**Figure 8:**
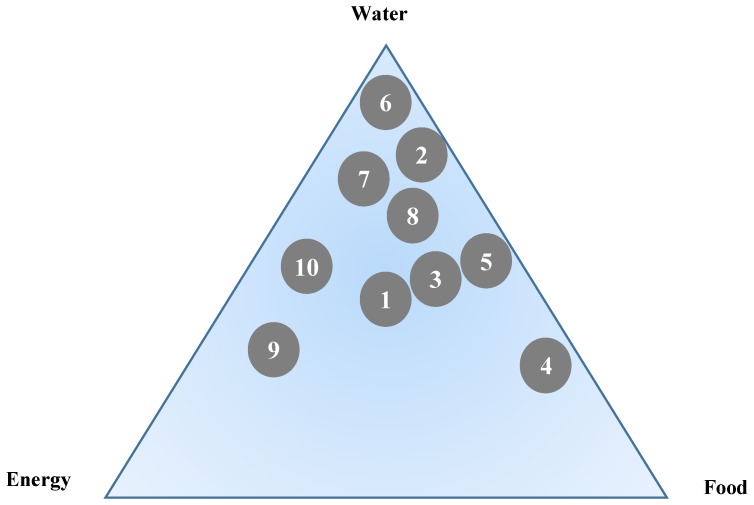
The conceptual location of references related to SDGs and water-energy-food nexus (please see Table 5 for the meaning of the number).

The top combination SDG 2-6-7 was precisely the “water-energy-food” (WEF) nexus, which has become a hot research issue in recent years, as climate change kept threatening the natural and human ecosystems [19]. The ten most relevant papers were chosen and examined through some qualitative points of view (Table 5). Water, energy, and food were designed as the three poles in Figure 8, and the location of each paper was determined based on the closeness to the three topics. As can be observed, most of the WEF nexus papers were inclined to address water issues in the SDG reference pool. From the management perspective, the security concept of the use of three sorts of resources led to the need of quantitative indicators, available data and integrated analytical tools for monitoring the status of resources [18,19,67,68,69,70,71]. Besides, ecosystems often were associated with water-energy-food nexus [69,72,73]. Furthermore, the nexus challenges were different in diverged regions [16,20,67,70,71], so contextual thinking for Water-Energy-Food nexus was needed. Overall, proper sectoral coordination would be the main factor of successfully managing WEF nexus [18,69,74]. Therefore, the exploration of strategies about creating the environment of cooperation and facilitating multi-stakeholder dialogue could be a promising direction.

Generally speaking, the creative works in academic and media communities were viewed as both the collective actions formed by relevant stakeholders toward achieving 2030 agenda and the knowledge translation strategies for expanding public participation. The SDG-related academic publications collected from the reference database “Scopus” and the SDG Lions lists of communication videos gather by Cannes Lion Festival with influential media networks. The current study aimed to investigate the possible consensus toward SDGs issues and elucidated the interactions of two SDGs through different methodologies, including content and network analysis. As a total of 1030 papers were sieved out from 1,105 academic publications from 2015 to 2018 and then examined. The videos analyzed in the study were the 687 entries of the 2018 “SDG Lions”.

It was found the number of academic papers linked to SDGs kept growing in recent years for each of the SDGs. The most common cited or related SDGs included SDG 3 (health and well-being), SDG 8 (economy and work), SDG 10 (equality), SDG 6 (water and sanitation), and SDG 4 (education) for the four years. In contrast, SDG 14 (life below water) and SDG 5 (gender equality) were the least related SDGs for the academia. While SDG 3 and SDG 10 shared the highest preference in the academia and the media, apparent gaps in preference of SDG 5 were observed between these two communities/industries, as SDG 5 was ranked the second place in the videos. If looking at the papers and videos in the same year of 2018, it was found that the relevant percentages of SDG 8 and SDG 9 (industry, innovation, and infrastructure) were much lower for the media industry, demonstrating a differentiated preference on these relatively “hard” topics.

A platform named “My World 2030” generate data about global people’s opinion on SDGs and ask a question like” What SDGs are worthy of immediate concern?” and found the top three SDGs people concerned in order were SDG 8, SDG 3, and SDG 5 [75]. That is, for the results obtained in either academia or the media, two-thirds of overlaps in the first three ranks could be found. SDG 3 was widely recognized as the most crucial SDG in common by the professionals and ordinary people. Consistent results were also found in the analysis of Austrian university publications with sustainability topics [31]. However, in some specified fields, SDG 3 was not emphasized as in these studies probably owing to specified sampling approaches, e.g., the study conducted by Salvia et al. [32]. Thus, multiple perspectives of representative samples could be needed to confirm key SDG issues.

The tied frequencies between the possible SDG pairs were also of significant concern in this study. It was found the SDG 3–10 pair had leading tied frequencies in both industries, which indicates health-related topics were frequently associated with reducing disparities or inequalities in different fields and scales. This finding also can be supported by the claim of bidirectional interactions between SDG 3–10 [9]. Conversely, the vast difference between the two industries happened in the linkage of SDG 3 and SDG 8 because it was ranked top two in academia but the least rank in the media. The degree centrality was employed to mark the importance of an SDG in the network composed of 17 SDGs. Generally speaking, SDG 3 and SDG 10 had powerful connections with other SDGs in both industries. Nevertheless, SDG 8 and SDG 5 had relatively secure connections with several SDGs for the academia and the media, respectively.

For demonstrating the whole picture of the information for a specific SDG, a graph capable of showing the relative scales of the tied frequencies and other parameters was designed. SDG 3 was selected as the core theme of the demonstration. Overall speaking, SDG 3 could be treated as a unifying theme when seeking to acquire evidenced-based knowledge in the integration of the SDGs, which was verified again in the analysis of possible combinations of three SDGs co-linked to a single paper. SDG 2-6-7 and SDG 3-10-16 were the most frequent combinations in the 319 papers with less than seven SDGs associated. SDG 3 was included eight times in the top ten list and four times in the top five list. The SDG 2-6-7 combination was precisely the “water-energy-food” (WEF) nexus, which has been widely studied as an emerging integrated sustainability-related topic in recent years.

To accomplish the mission of sustainable development, implementing SDGs in an integrated way is needed [1,7,8,17,76]. Integration does mean not only effective incorporation of all SDGs but also interactive collaborations among different stakeholders, e.g., academia, and media, and other professionals. Caiado et al. indicated that one of the challenges in implementing the SDGs was to promote cooperation among parties [77]. Boutilier found the social capital and issues of interest would affect the potential of cooperation [78]. In this sense, the focused SDGs and common ones for the academia and the media identified in this study would represent the pivot issues for cross-boundary or interdisciplinary cooperation. Endo et al. investigated the participants of a WEF nexus platform and found that more than half of them were research organization in the academia, but only less than 1.5% of media registered [20]. A wide range of engaged stakeholder (including the media) can benefit knowledge translation and transmission toward the public [30]. Moreover, enhanced stakeholder relations create some advantages, comprising the reduction of resistance and commitment to resolutions [79]. Thus, the current study cast the first stone in the exploration of key stakeholders’ perspectives, finding the key SDGs, SDG pairs of interest of the academia and the media, followed by network analysis for examining the critical interrelationships among them. This can be a good base for establishing strategic plans and carrying out integrated implementations of SDGs with the academia, the media, and other stakeholders involved.

## 4. Conclusions

Creative works in academic and media communities were viewed as both collective actions undertaken by relevant stakeholders toward achieving 2030 agenda and knowledge translation strategies for expanding public participation. The current study aimed to investigate the possible consensus toward SDGs issues and elucidated the interactions of two SDGs through different methodologies, including content and network analysis.

To accomplish the mission of transforming our world towards sustainable development, implementing SDGs in an integrated way is needed [1,7,8,17,76]. Here integration means not only effective incorporation of all SDGs but also interactive collaborations among different stakeholders, e.g., academia, and media, and other professionals. The co-occurrence of two or three SDGs for a paper or video could offer vital information for effective incorporation among the SDGs, while an understanding toward the perspectives of SDG-related stakeholders could give feedback when consulting the priority of implementing SDGs.

The current study was funded by the Ministry of Science of Technology in Taiwan, belonging to the theme of “science, technology, communication, and society”. The voices of the communication industry and the lens of the broader audience toward sustainable development related issues were emphasized. These are especially important because the perspective of the media have been overlooked in the formal SD-related discussions [20,30].

The present study individually and holistically examined the impact of each SDG from the viewpoints of both academic and communication community whose works serve diverged audiences. The academic publications informed policymakers with evidence-based knowledge and information through a top-down route. In contrast, the informative videos spread understandable messages to the general public in a bottom-up style. The academia offers supporting power for efficient or effective management of SD-related issues, while the impact of media may be linked to petition to acquire the right of decision on public affairs, which is a key component of governance for SD. In the process of SDG-related policy-making, these two patterns of power could generate synergy effects as long as the common grounds were found. Several studies found that promoting cooperation among parties was challenging [77,78,79]. Boutilier claimed the social capital and issues of interest would affect the potential of cooperation [78]. In this sense, the focused SDGs and common ones for the academia and the media identified in this study would represent the pivot issues for cross-boundary or interdisciplinary cooperation.

The current study found the issues related to human health and well-being (SDG 3) are widely concerned in the perspectives of both the academia and the communication community as SDG 3 seems to have an immediate and apparent influence on human. These are especially true as a contrast of the general environmental issues, which tend to be treated as public affairs instead of personal issues as long as there are no direct and prompt impacts on people. In other words, SDG 3 has shorter physical and psychological distances for most people, making it popular in terms of academic research and public communication. Besides, the most frequent pair of SDG 3 and SDG 10 (reduce inequality) could be applied for relevant national policies with potential impacts on the health and well-being of people in different social status. These also remind us that choices of health-related services, including medical care and food/water, would be linked to socio-economic factors of people. This is the reason why SDG 3 and SDG 10 need to be taken into consideration at the same time. Moreover, the multiple linkages among SDG 3, SDG 10, and SDG16 could extend the discussion into what kind of institutions could secure poor people’s health. To sum up, the current study cast the first stone in the exploration of key stakeholders’ perspectives, finding the key SDGs, SDG pairs and combinations of interest of the academia and the media, followed by network analysis for examining the critical interrelationships among them. These can be a good base for establishing strategic plans and carrying out integrated implementations of SDGs with the academia, the media, and other stakeholders involved.

## Figures and Tables

**Table 1:** The guidelines for categorizing SDG-related publications.

SDG	Relevant Keywords
1. No poverty	poverty; *poor
2. Zero hunger	hung*; food; agricult*; *nutriti*; “food energy”
3. Good health and well-being	health*; mortality; death*; disease*; illness*
	medicine*; vaccine*; “world health organization”; WHO
4. Quality education	educat*; learn*; school*; train*; knowledge; skill*; teach*
5. Gender equality	“gender equality”; wom?n; girl*; femal*; feminis*; marriage; unpaid
6. Clean water and sanitation	*water*; hydro*; aqua*; sanitation*; hygien*
7. Affordable and clean energy	*energy; *fuel; electri*; biomass; *power
8. Decent work and economic growth	“decent work”; “decent job”; *employ*; worker*; labour; labor; *econom*; “economic growth”;
	“financial institution*”; “financial perform*”; “corporate social responsibility”; CSR; business
9. Industry, innovation and infrastructure	Infrastructure; industr*; innovation; research*; internet; technology
	“Information and communications technology”; ICT
10. Reduced inequalities	*equal*; inclus*; “protect* polic*”; “developing countr*”; “least developed countr*”;
	“low?income countr*”; mobility; migra*; “world trade organization”; WTO
11. Sustainable cities and communities	city; cities; urban*; settlement*; housing; slum*; transport*; heritage; “public space*”; building*; “disaster risk management”; “air quality”
12. Responsible consumption and production	consumption; consumer; production; product*; waste*; chemi*; reuse; recycle*;
	“corporate social responsibility”; CSR; subsid*; “green economy”; “circular economy”;
	“low-carbon economy”; “green product”; “green growth”; “clean growth”; “environmental tax*”
13. Climate action	climat*
14. Life below water	ocean*; mari*; sea*; coast*; *fish*; “life ? water”
15. Life on land	*land*; ecosystem*; *forest*; terrestrial; biodiversity; “biological diversity”; desertification
16. Peace, justice and strong institutions	peace*; violen*; war; just*; institution*; law*; crim*; *legal*; legislat*; act*
17. Partnerships for the goals	global*; world; internation*; cooperat*; partnership*; trade; export

Note. The Boolean search was used to expand the amount of publication in the resulting pool of the Scopus database. Quotation mark means the connected keywords as one unit and to be searched together; asterisk mark means the front and/or back of the keyword might insert letter(s); question mark means the middle of a word or a phrase insert a letter and/or a word.

**Table 2:** The crosstabulation of SDG-paired works in academia and media.

F	SDG 1	SDG 2	SDG 3	SDG 4	SDG 5	SDG 6	SDG 7	SDG 8	SDG 9	SDG 10	SDG 11	SDG 12	SDG 13	SDG 14	SDG 15	SDG 16	SDG 17
SDG 1	—																
	—																
SDG 2	121	—															
	*2*	—															
SDG 3	143	127	—														
	*1*	*6*	—														
SDG 4	116	109	152	—													
	*3*	*1*	*3*	—													
SDG 5	104	91	124	107	—												
	*1*	*0*	*3*	*1*	—												
SDG 6	107	124	136	104	93	—											
	*0*	*0*	*2*	*0*	*0*	—											
SDG 7	104	118	107	96	87	125	—										
	*1*	*0*	*2*	*0*	*0*	*1*	—										
SDG 8	126	129	142	130	109	117	110	—									
	*4*	*1*	*0*	*3*	*3*	*0*	*0*	—									
SDG 9	103	116	113	115	88	110	112	125	—								
	*0*	*0*	*1*	*0*	*0*	*0*	*1*	*1*	—								
SDG 10	127	107	160	110	112	109	99	127	106	—							
	*7*	*0*	*14*	*7*	*39*	*0*	*1*	*3*	*1*	—							
SDG 11	101	108	112	98	89	115	99	112	112	102	—						
	*0*	*1*	*5*	*1*	*1*	*3*	*3*	*2*	*4*	*6*	—						
SDG 12	94	111	107	97	87	104	108	118	118	93	99	—					
	*0*	*2*	*4*	*0*	*0*	*3*	*4*	*1*	*2*	*0*	*4*	—					
SDG 13	99	118	112	99	89	112	116	109	106	103	111	105	—				
	*0*	*1*	*1*	*0*	*0*	*3*	*1*	*0*	*0*	*0*	*2*	*3*	—				
SDG 14	94	95	97	89	83	95	94	98	92	90	90	91	98	—			
	*0*	*0*	*1*	*0*	*0*	*3*	*0*	*0*	*2*	*0*	*1*	*5*	*2*	—			
SDG 15	106	127	110	97	85	121	114	121	110	98	102	110	130	117	—		
	*0*	*0*	*1*	*0*	*0*	*2*	*1*	*0*	*1*	*0*	*3*	*3*	*4*	*1*	—		
SDG 16	107	98	132	113	103	99	96	114	97	125	95	91	101	90	100	—	
	*1*	*0*	*3*	*2*	*3*	*0*	*0*	*1*	*0*	*11*	*3*	*0*	*0*	*0*	*0*	—	
SDG 17	95	92	126	106	85	94	89	106	96	112	88	91	94	81	90	113	—
	*1*	*3*	*6*	*1*	*0*	*2*	*1*	*1*	*2*	*6*	*3*	*3*	*4*	*2*	*3*	*4*	—
A	1747	1791	2000	1738	1536	1765	1674	1893	1719	1780	1633	1624	1702	1494	1738	1674	1558
M	*21*	*17*	*53*	*22*	*51*	*19*	*16*	*20*	*15*	*95*	*42*	*34*	*21*	*17*	*19*	*28*	*42*

Note. 2018 SDG Lions videos in lower cells (italic) and academic publications for four years in upper cells. F means the frequency of two SDGs; A means the sum of specific SDG pairs in the same column among academic papers; M means the sum of specific SDG pairs in the same column among media videos.

**Table 3:** The top ten SDGs with highest degree centralities in the network with 136 SDG pairs.

Rank	1	2	3	4	5	6	7	8	9	10
Academic publications	SDG 3	SDG 8	SDG 10	SDG 2	SDG 6	SDG 1	SDG 15	SDG 4	SDG 9	SDG 13
Degree Centrality	0.781	0.739	0.695	0.700	0.689	0.682	0.679	0.679	0.671	0.665
Media	SDG 10	SDG 3	SDG 5	SDG 11	SDG 17	SDG 12	SDG 16	SDG 4	SDG 1	SDG 13
Degree Centrality	0.152	0.085	0.082	0.067	0.067	0.054	0.045	0.035	0.034	0.034

**Table 4:** The top most frequent list of SDG combinations with three SDGs co-linked in a paper.

	SDG#	1	2	3	4	5	6	7	8	9	10	11	12	13	14	15	16	17	Total Frequency
Combination																			
2, 6, 7			*				*	*											14
3, 10, 16				*							*						*		14
1, 3, 10		*		*							*								13
3, 4, 10				*	*						*								13
1, 3, 4		*		*	*														13
3, 4, 8				*	*				*										12
3, 10, 17				*							*							*	11
2, 13, 15			*											*		*			11
3, 5, 10				*		*					*								11
1, 2, 3		*	*	*															10
# of inclusion		3	3	8	3	1	1	1	1	0	5	0	0	1	0	1	1	1	

Note. The symbol # indicated which SDG was discussed; and the symbol * indicated the presence of a certain SDG.

**Table 5:** The list of references related to SDGs and water-energy-food nexus.

ID	Year	Author	Title	Journal
1	2018	Albrecht et al.	The water-energy-food nexus: A systematic review of methods for nexus assessment [18]	*Environmental Research Letters*
2		Avellán et al.	Considering resources beyond water: Irrigation and drainage management in the context of the Water-Energy-Food nexus. [72]	*Irrigation and Drainage*
3		Mainali et al.	Evaluating synergies and trade-offs among sustainable development goals (SDGs): Explorative analyses of development paths in south Asia and Sub-Saharan Africa [70]	*Sustainability* (Switzerland)
4		Nhamo et al.	The water-energy-food nexus: Climate risks and opportunities in southern Africa. [16]	*Water* (Switzerland)
5		Nhemachena et al.	Measuring baseline agriculture-related sustainable development goals index for southern Africa. [71]	*Sustainability* (Switzerland)
6	2017	Giupponi & Gain	Integrated spatial assessment of the water, energy and food dimensions of the sustainable development goals. [68]	*Regional Environmental Change*
7		Liu et al.	Challenges in operationalizing the water–energy–food nexus. [19]	*Hydrological Sciences Journal*
8		Pahl-Wostl	Governance of the water-energy-food security nexus: A multi-level coordination challenge. [69]	*Environmental Science and Policy*
9	2016	Rasul	Managing the food, water, and energy nexus for achieving sustainable development goals in South Asia. [74]	*Environmental Development*
10		Yillia	Water-energy-food nexus: Framing the opportunities, challenges and synergies for implementing the SDGs. [73]	*Osterreichische Wasser-Und Abfallwirtschaft*
